# Tranexamic acid in pediatric trauma: why not?

**DOI:** 10.1186/cc13965

**Published:** 2014-07-02

**Authors:** Suzanne Beno, Alun D Ackery, Jeannie Callum, Sandro Rizoli

**Affiliations:** 1 The Hospital for Sick Children, 555 University Avenue, Toronto Ontario M5G 1X8, Canada; 2 St Michael’s Hospital, 30 Bond Street, Toronto, Ontario M5B 1 W8, Canada; 3 Sunnybrook Health Sciences Centre, 2075 Bayview Avenue, Room B2 04, Toronto, Ontario M4N 3M5, Canada

## Abstract

Trauma is a leading cause of death in pediatrics. Currently, no medical treatment exists to reduce mortality in the setting of pediatric trauma; however, this evidence does exist in adults. Bleeding and coagulopathy after trauma increases mortality in both adults and children. Clinical research has demonstrated a reduction in mortality with early use of tranexamic acid in adult trauma patients in both civilian and military settings. Tranexamic acid used in the perioperative setting safely reduces transfusion requirements in children. This article compares the hematologic response to trauma between children and adults, and explores the potential use of tranexamic acid in pediatric hemorrhagic trauma.

## Introduction

Trauma is a leading cause of mortality in the pediatric population. In 2008, the American Academy of Pediatrics noted that trauma accounts for more deaths than all other causes combined [1]. Bleeding remains the most preventable cause of death after trauma. A major recent advance was the recognition that coagulation defects are greatly responsible for the disproportional mortality caused by bleeding. Managing coagulation defects has become a forefront issue in trauma and novel strategies proposed include hemostatic resuscitation, adoption of massive transfusion protocols and other innovations [2], most with little evidence to support them. The exception is the proposal to administer tranexamic acid (TXA) to bleeding adult trauma patients.

To date, no medical treatment has been shown to reduce mortality in the setting of pediatric trauma; however, this evidence does exist in adults. Bleeding and coagulopathy due to trauma are associated with mortality in both adults and children. Clinical research has demonstrated a reduction in trauma-related mortality with early use of TXA in adult patients in both civilian and military settings [3,4]. In adult patients with hemodynamic instability and ongoing bleeding, TXA is expected to save 1 in 67 lives [3]. There is no scientific or biological reason to suggest that a similar mortality benefit will not be seen in pediatric trauma. We feel the incorporation of TXA into pediatric trauma management has the potential to also significantly reduce mortality in children and youths, without increasing adverse events. This review explores the hematologic differences and similarities between injured children and adults, and the idea that TXA may be a novel and promising treatment in pediatric hemorrhagic trauma despite the current lack of evidence for its use in this setting.

TXA is an antifibrinolytic that reversibly binds to plasminogen at the lysine binding site, thus preventing the binding of plasmin (ogen) to fibrin and the subsequent degradation of fibrin [5]. It is a generic, inexpensive medication used to prevent fibrin breakdown and reduce bleeding in various clinical settings (including orthopedic and cardiovascular surgery, post-partum hemorrhage, gastrointestinal hemorrhage, epistaxis, certain ophthalmologic conditions and other obstetric/gynecologic emergencies) [6-9]. The cost per adult trauma patient is a mere Canadian $11.80 (2 grams), with a cost per life saved of Canadian $790.60 [3]. Patients are particularly susceptible to hyperfibrinolysis after traumatic injury, with 57% having moderate hyperfibrinolysis as detected by plasmin-antiplasmin complexes, which theoretically could be inhibited by TXA [10].

## Evidence for tranexamic acid in adult trauma

In 2010, CRASH-2 (Clinical Randomization of an Antifibrinolytic in Significant Haemorrhage-2) demonstrated that TXA safely reduces the risk of death in adult (age ≥16 years) trauma. This was the first study to show a medical treatment for trauma and a reduction in mortality. This randomized placebo-controlled trial involved 20,211 patients in 40 countries. Adult patients with unstable vitals (systolic blood pressure <90 mmHg and/or heart rate >110 beats per minute, or both) or a high clinical suspicion for significant hemorrhage were randomized to TXA versus placebo. The primary outcome, in-hospital mortality at 4 weeks, showed a significant reduction in risk of death due to bleeding for the TXA group (risk of death 0.85; 95% confidence interval 0.76 to 0.96; *P* = 0.004, number needed to treat (NNT) 121) and all-cause mortality for the overall population (risk of death 0.91; 95% confidence interval 0.85 to 0.97; *P* = 0.0035, NNT 67). TXA appeared to have the greatest impact on reduction of death caused by bleeding in the severe shock group (admission systolic blood pressure ≤75 mmHg) if administered within the first hour from injury. Further sub-analysis of CRASH-2 showed that mortality benefit was greatest when given within 3 hours of the injury, with suggested increased risk of death caused by bleeding, but no difference in all-cause mortality, if given later [3,11].

The CRASH-2 trial administered 1 g of TXA over 10 minutes followed by 1 g over 8 hours [3]. Adverse events have not been published with this dose, but reports exist from other patient populations when much larger doses are given. In cardiovascular bypass surgery, extremely high doses (>100 mg/kg) of TXA appear to increase the risk of perioperative seizures [12-15]. There are also reports of TXA rapid infusion hypotension, TXA-induced cerebrovascular infarctions and even a single case report of TXA-induced toxic epidermal necrolysis [16-18]. Vascular occlusive events were included in the secondary outcomes in the CRASH-2 trial, but there were no significant differences between TXA and control groups with respect to arterial and venous thromboembolic events [3]. Systematic reviews have failed to find a concern regarding adverse events [19-21].

CRASH-2 was a landmark trial involving a large and diverse study population in many different healthcare settings. It provides high-level evidence for the widespread global use of TXA in adult trauma patients with significant hemorrhage. Conversely, the fact that the majority of sites were in low- and middle-income countries has drawn criticism for CRASH-2. Concerns include an over-reliance on results that may not be accurate for patients cared for in developed comprehensive trauma systems with lower preventable death rates through access to blood products, massive transfusion protocols and definitive surgical care. However, Ker and colleagues [22] found no compelling evidence that geography varied the effect of TXA on death due to bleeding when jointly analyzing CRASH-2 trial data, World Health Organization mortality data, and data from a systematic review. Lastly, the impressive rarity of thrombotic complications seen in CRASH-2 has sparked concerns that the possible risk of thromboembolism may have been underappreciated and might well be higher in hospitals where these complications would be more actively sought [23]. While current evidence does not support this theory, the focus on thromboembolism merits further research.

More recent *post hoc* analysis showed that administering TXA to bleeding trauma patients can save an estimated 755 ‘life-years’ per 1,000 trauma patients in the United Kingdom, with the incremental cost per ‘life-year’ gained by administering TXA being $64 [24]. Another retrospective observational study published in 2011, the Military Application of TXA in Trauma Emergency Resuscitation study, demonstrated an absolute reduction in both in-hospital and all-cause mortality at 48 hours for the TXA group of 7.6% and 6.5%, respectively. The benefit (13.7%) appeared greatest in the groups receiving massive transfusion (estimated NNT 7) [4]. Unlike the pragmatic global approach that CRASH-2 took, this study population included young healthy soldiers receiving advanced first world trauma care for whom this significant reduction in mortality with TXA was also found. As a result, standard of care in many parts of the world involves administering TXA to adult trauma patients with suspected hemorrhage.

Future studies looking at TXA use in subarachnoid hemorrhage are already underway and a CRASH-3 trial is being organized to evaluate the utility and safety of TXA in isolated traumatic brain injury (TBI) [25].

## Tranexamic acid use in pediatrics to date

As in adults, TXA has been well studied in many non-traumatic pediatric conditions. The numerous studies in adult and pediatric populations demonstrate similar benefit from TXA in the perioperative setting. TXA has successfully reduced blood loss and transfusion requirements with a variety of dosing regimens in pediatric cardiac surgery, scoliosis surgery, and craniosynostosis repair [26-28]. It has an important role in managing certain menstrual disturbances and congenital bleeding disorders, and is effective in treating traumatic hyphema in children [29]. The adverse effects of TXA in pediatrics are very rare but include gastrointestinal effects, hypotension with rapid intravenous injection, dizziness, headache, muscle pain and spasms, and postoperative convulsions in children receiving high doses during cardiac surgery. The potential risk of thrombosis has not been identified with large doses in pediatric cardiac and spinal surgery, and the lower risk in the pediatric population overall makes this a less concerning issue [21,27,28].

## Trauma: differences and similarities between adults and children

In adult trauma, hemorrhage is the second overall cause of death, first preventable cause and first cause of mortality after arrival to hospital [30]. Recently, there is increasing appreciation for ‘early trauma-associated coagulopathy’, an entity independent of fluid resuscitation and transfusion. It is estimated that 25% of adult trauma victims are coagulopathic upon arrival to the hospital, and when thromboelastography (TEG®) and rotational thromboelastometry (ROTEM®) are used diagnostically, up to 6% of these patients already have massive hyperfibrinolysis. Evidence further shows that up to 60% may have less intense hyperfibrinolysis diagnosed by plasmin-antiplasmin complexes, tissue plasminogen activator and D-dimer levels [31]. It stands to reason, therefore, that the antifibrinolytic drug TXA, both theoretically and following high level evidence, now has a clear role in the injured and bleeding adult.

Children follow a slightly different pattern of trauma than adults, with variability existing due to environmental factors and the broad anatomic, physiologic and developmental age spectrum that encompasses pediatric care. Systolic blood pressures in children vary by age, and hypotension is often seen only once an uncompensated shock state exists (Table 1). The proportion of blunt to penetrating trauma is higher in children, but the leading cause of mortality is also traumatic brain injury, a condition known to be associated with coagulopathy in both pediatric and adult populations [32,33]. While the incidence of death from hemorrhage with traumatic injuries has not been described in children, hemorrhage remains the most common cause of death in pediatric solid organ injuries, and blunt thoracoabdominal trauma resulting in hemorrhage is the second leading cause of traumatic death in children [34].

**Table 1:** Criteria for the use of tranexamic acid in pediatric trauma

**Immediate need for transfusion, with any one of the following indicating severe shock**^**a**^	
•	Systolic blood pressure low (<80 mmHg <5 years and <90 mmHg ≥5 years)
•	Poor blood pressure response to crystalloid 20–40 ml/kg
•	Obvious significant bleeding

^a^The Hospital for Sick Children Massive Hemorrhage Protocol for the use of tranexamic acid in pediatric trauma. April 2014.

**Table 2:** Tranexamic acid dosing in pediatric trauma

**Age**	**Loading dose (administer within 3 hours)**	**Subsequent dose**
≥12 years::adult protocol	1 g intravenously over 10 minutes	1 g intravenous infusion over 8 hours
<12 years	15 mg/kg intravenously over 10 minutes (maximum dose 1 g)	2 mg/kg/hr intravenous infusion over 8 hours or until bleeding stops

The Hospital for Sick Children Massive Hemorrhage Protocol for the use of tranexamic acid in pediatric trauma. April 2014. Adapted from Royal College of Paediatrics and Child Health: Evidence statement - Major trauma and the use of tranexamic acid in children [39].

## The coagulopathic response to trauma in children

Levels of coagulation factors vary by age and maturation, as does the tendency to clot or bleed, leaving pediatric trauma patients more susceptible to bleeding when coagulant maturity lags behind its anticoagulant counterpart. Given the high rates of TBI in the pediatric population and its possible association with hyperfibrinolysis and coagulopathy, the coagulation status of injured children has been addressed in the literature. Coagulopathy in both pediatric and adult head trauma is well described, and its published incidence ranges from 15% to 87% [32-34]. In pediatric blunt trauma, coagulopathy was reported in 28% of patients by Holmes and colleagues [35], with significant coagulopathy occurring in 6% of children and associated with a Glasgow Coma Scale score ≤13, low systolic blood pressure, open/multiple fractures, and major tissue wounds. Hendrickson and colleagues [36] described coagulopathy in pediatric trauma patients specifically requiring transfusion support using a combined retrospective review and prospective analysis of 102 children (mean age of 6 years, injury severity score of 22 and Glasgow Coma Scale score of 7) presenting to an emergency department over a 4-year period. Overall, 77% of the patients were coagulopathic on arrival to the emergency department, and abnormal coagulation parameters remained strongly associated with mortality even after adjustment for injury severity score. Coagulopathy was concluded to be prevalent, possibly even more common than in adults, in pediatric trauma patients ill enough to require a transfusion [36]. An evaluation of the role of early coagulopathy and shock as independent predictors of mortality in children with traumatic injuries treated at combat support hospitals in Iraq and Afghanistan described results similar to those previously reported for adults, with 27% of the children demonstrating coagulopathy on admission, and a higher injury severity score predicting increased coagulopathy, shock, and ultimately mortality [37].

The role of hyperfibrinolysis in pediatric trauma patients is not as well established as in adults, although hypofibrinogenemia has been reported in up to 52% of children requiring transfusion support [36]. The experience of using TEG® and ROTEM® technology for pediatric trauma has been published in case reports and a recent retrospective review utilizing rapid thrombelastography (rTEG) in 86 children [38]. While not specifically addressing hyperfibrinolysis, this paper demonstrated that rTEG provided valuable goal-directed hemostatic resuscitation data for critically injured children. The available literature implies that injured children and adults respond similarly with regards to early coagulopathy and its association with certain injuries such as TBI that promote hyperfibrinolysis in the body, the latter being an important area of research requiring further exploration.

## Tranexamic acid for pediatric trauma

The basis for using TXA in children would appear to be nearly identical to that for adults, if not more intuitive. Since children generally have healthier vascular systems than adults, and with the main concern being thromboembolism, there is no clear reason for enforcing a lower age limit for this drug. At least in adolescents, where we can confidently presume their coagulation system has matured and their injury patterns resemble those of adults, the role of TXA should be adopted immediately. In fact, five participants in CRASH-2 were inadvertently enrolled and later discovered to be under 16 years of age [3]. The Royal College of Paediatrics and Child Health in the United Kingdom issued an Evidence statement in November 2012 entitled ‘Major trauma and the use of TXA in children’ that proposes the use of TXA for all children [39]. The dosing consensus reached utilizes the adult protocol for a 1 g loading dose over 10 minutes within the first 3 hours post-injury, followed by a 1 g infusion over 8 hours for children 12 years of age and older. For children under 12 years of age, the loading dose was pragmatically estimated at 15 mg/kg (maximum dose 1 g) followed by an infusion of 2 mg/kg/hour for at least 8 hours or until the bleeding stops [39] (Table 2). The doses of TXA used for trauma are significantly lower than the published doses in use for pediatric cardiac, spinal and craniofacial surgery, and even at these doses adverse events are rare. The emerging concern of post-administration seizures has not been reported for the dose used in trauma.

## Use of tranexamic acid in pediatric hemorrhagic trauma

According to best available evidence in 2014, TXA significantly decreases mortality in bleeding trauma patients 16 years of age and older without significantly increasing prothrombotic complications if administered within 3 hours of injury. Currently, there is no evidence of benefit in patients with TBI, and results from CRASH-3 should guide treatment in this area. Early treatment with TXA is recommended in adult trauma patients at risk for significant hemorrhage, patients being transfused, and particularly those requiring massive transfusion or who have an elevated baseline risk of death [40].

While the evidence specifically for TXA in pediatric trauma is not yet available, we feel that TXA should be considered for use in adolescent trauma patients in the same dosing regimen and indications as used in adults. Given the consistency in coagulation response to tissue injury across age spectrums, we feel that young children with hemodynamic instability and ongoing risk for hemorrhage would also benefit from TXA while recognizing this specifically as an area in need of further research. The adoption and use of this inexpensive and cost-effective therapy may reach only a small percentage of pediatric trauma patients. However, without anticipated risk, in children requiring transfusion support following trauma, TXA plausibly holds similar or greater benefit for mortality reduction than has already been demonstrated in adults. Denying injured children TXA due to the lack of pediatric trauma trial evidence in this indication is likely shortsighted and unnecessary given the ample clinical evidence in other pediatric settings, the excellent safety record of the drug, and the clear mortality benefit seen in adult trauma.

## Abbreviations

CRASH-2: Clinical randomization of an antifibrinolytic in significant haemorrhage-2; NNT: Number needed to treat; ROTEM®: Rotational thromboelastometry; rTEG: Rapid thrombelastography; TBI: Traumatic brain injury; TEG®: Thromboelastography; TXA: Tranexamic acid.

## Competing interests

The authors declare that they have no competing interests.

## References

[B1] American Academy of Pediatrics, Pediatric Orthopedic Society of North America Policy statement: management of pediatric trauma Pediatrics 2008 121 849 85418381551

[B2] Dzik WH Blajchman MA Fergusson D Hameed M Henry B Kirkpatrick AW Korogyi T Logsetty S Skeate RC Stanworth S MacAdams C Muirhead B Clinical review: Canadian National Advisory Committee on Blood and Blood Products - massive Transfusion Consensus Conference 2011: report of the panel Crit Care 2011 15 242 10.1186/cc10498 22188866 PMC3388668

[B3] Shakur H Roberts I Bautista R Caballero J Coats T Dewan Y El-Sayed H Gogichaishvili T Gupta S Herrera J Hunt B Iribhogbe P Izurieta M Khamis H Komolafe E Marrero MA Mejia-Mantilla J Miranda J Morales C Olaomi O Olldashi F Perel P Peto R Ramana PV Ravi RR Yutthakasemsunt S CRASH-2 trial collaborators Effects of tranexamic acid on death, vascular occlusive events, and blood transfusion in trauma patients with significant haemorrhage (CRASH-2): a randomised, placebo-controlled trial Lancet 2010 376 23 32 20554319 10.1016/S0140-6736(10)60835-5

[B4] Morrison JJ Dubose JJ Rasmussen TE Midwinter MJ Military application of tranexamic acid in trauma emergency resuscitation (MATTERS) study Arch Surg 2012 147 113 119 10.1001/archsurg.2011.287 22006852

[B5] Okamoto S Hijikata-Okunomiya A Wanaka K Okada Y Okamoto U Enzyme controlling medicines: introduction Semin Thromb Hemost 1997 23 493 501 10.1055/s-2007-996127 9469621

[B6] Henry D Carless P Fergusson D Laupacis A The safety of aprotinin and lysine-derived antifibrinolytic drugs in cardiac surgery: a meta-analysis CMAJ 2009 180 183 193 10.1503/cmaj.081109 19050037 PMC2621296

[B7] Kagoma YK Crowther MA Douketis J Bhandari M Eikelboom J Lim W Use of antifibrinolytic therapy to reduce transfusion in patients undergoing orthopedic surgery: a systematic review of randomized trials Thromb Res 2009 123 687 696 10.1016/j.thromres.2008.09.015 19007970

[B8] Naoulou B Tsai MC Efficacy of tranexamic acid in the treatment of idiopathic and non-functional heavy menstrual bleeding: a systematic review Acta Obstet Gynecol Scand 2012 91 529 537 10.1111/j.1600-0412.2012.01361.x 22229782

[B9] Gluud LL Klingenberg SL Langholz SE Systematic review: tranexamic acid for upper gastrointestinal bleeding Aliment Pharmacol Ther 2008 27 752 758 10.1111/j.1365-2036.2008.03638.x 18248659

[B10] Raza I Davenport R Rourke C Platton S Manson J Spoors C Khan S De’Ath HD Allard S Hart DP Pasi KJ Hunt BJ Stanworth S MacCallum PK Brohi K The incidence and magnitude of fibrinolytic activation in trauma patients J Thromb Haemost 2013 11 307 314 10.1111/jth.12078 23176206

[B11] Roberts I Shakur H Afolabi A Brohi K Coats T Dewan Y Gando S Guyatt G Hunt BJ Morales C Perel P Prieto-Merino D Woolley T CRASH-2 trial collaborators The importance of early treatment with tranexamic acid in bleeding trauma patients: an exploratory analysis of the CRASH-2 randomised controlled trial Lancet 2011 377 1096 1101 21439633 10.1016/S0140-6736(11)60278-X

[B12] Murkin JM Falter F Granton J Young B Burt C Chu M High-dose tranexamic acid is associated with nonischemic clinical seizures in cardiac surgical patients Anesth Analg 2010 110 350 353 10.1213/ANE.0b013e3181c92b23 19996135

[B13] Kalavrouziotis D Voisine P Mohammadi S Dionne S Dagenais F High-dose tranexamic acid is an independent predictor of early seizure after cardiopulmonary bypass Ann Thorac Surg 2012 93 148 154 10.1016/j.athoracsur.2011.07.085 22054656

[B14] Manji RA Grocott HP Leake J Ariano RE Manji JS Menkis AH Jacobsohn E Seizures following cardiac surgery: the impact of tranexamic acid and other risk factors Can J Anaesth 2012 59 6 13 10.1007/s12630-011-9618-z 22065333

[B15] Koster A Borgermann J Zittermann A Lueth JU Gillis-Januszewski T Schirmer U Moderate dosage of tranexamic acid during cardiac surgery with cardiopulmonary bypass and convulsive seizures: incidence and clinical outcome Br J Anaesth 2012 59 6 13 10.1093/bja/aes310 22986419

[B16] Trauma and severe bleeding. Tranexamic acid within one hour to reduce mortality Prescrire Int 2013 22 189 190 23951599

[B17] Ross J Salman RA The frequency of thrombotic events among adults given antifibrinolytic drugs for spontaneous bleeding: systematic review and meta-analysis of observational studies and randomized trials Curr Drug Saf 2012 7 44 54 10.2174/157488612800492744 22663958

[B18] Pretel IM Marques ML Aguado GL Idoate Gastearena MA Tranexamic acid-induced toxic epidermal necrolysis Ann Pharmacother 2013 47 e16 10.1345/aph.1R637 23447480

[B19] Ker K Edwards P Perel P Shakur H Roberts I Effect of tranexamic acid on surgical bleeding: systematic review and cumulative meta-analysis BMJ 2012 344 e3054 10.1136/bmj.e3054 22611164 PMC3356857

[B20] Henry DA Carless PA Moxey AJ O’Connell D Stokes BJ Fergusson DA Ker K Anti-fibrinolytic use for minimising perioperative allogeneic blood transfusion Cochrane Database Syst Rev 2011 3 CD001886 21412876 10.1002/14651858.CD001886.pub4 PMC4234031

[B21] Faraoni D Goobie SM The efficacy of antifibrinolytic drugs in children undergoing noncardiac surgery: a systematic review of the literature Anesth Analg 2014 118 628 636 10.1213/ANE.0000000000000080 24557107

[B22] Ker K Junko K Perel P Edwards P Shakur H Roberts I Avoidable mortality from giving tranexamic acid to bleeding trauma patients: an estimation based on WHO mortality data, a systematic literature review and data from the CRASH-2 trial BMC Emerg Med 2012 12 3 10.1186/1471-227X-12-3 22380715 PMC3314558

[B23] Pusateri AE Weiskopf RB Bebarta V Butler F Cestero RF Chaudry IH Deal V Dorlac WC Gerhardt RT Given MB Hansen DR Hoots WK Klein HG Macdonald VW Mattox KL Michael RA Mogford J Montcalm-Smith EA Niemeyer DM Prusaczyk WK Rappold JF Rassmussen T Rentas F Ross J Thompson C Tucker LD US DoD Hemorrhage and Resuscitation Research and Development Steering Committee Tranexamic acid and trauma: current status and knowledge gaps with recommended research priorities Shock 2013 39 121 126 10.1097/SHK.0b013e318280409a 23222525

[B24] Guerriero C Cairns J Perel P Shakur H Roberts I Cost-effectiveness analysis of administering tranexamic acid to bleeding trauma patients using evidence from the CRASH-2 trial PLoS One 2011 6 e18987 10.1371/journal.pone.0018987 21559279 PMC3086904

[B25] Dewan Y Komolafe EO Mejia-Mantilla JH Perel P Roberts I Shakur H CRASH-3 Collaborators CRASH-3-tranexamic acid for the treatment of significant traumatic brain injury: study protocol for an international randomized, double-blind, placebo-controlled trial Trials 2012 13 87 10.1186/1745-6215-13-87 22721545 PMC3481366

[B26] Basta MN Stricker PA Taylor JA A systematic review of the use of antifibrinolytic agents in pediatric surgery and implications for craniofacial use Pediatr Surg Int 2012 28 1059 1069 10.1007/s00383-012-3167-6 22940882

[B27] McLeod LM French B Flynn JM Dormans JP Keren R Antifibrinolytic use and blood transfusions in pediatric scoliosis surgeries performed at US children’s hospitals J Spinal Disord Tech 2013 [Epub ahead of print] 10.1097/BSD.0b013e3182a22a54 24091932

[B28] Grassin-Delyle S Couturier R Abe E Alvarez JC Devillier P Urien S A practical tranexamic acid dosing scheme based on population pharmacokinetics in children undergoing cardiac surgery Anesthesiology 2013 118 853 862 10.1097/ALN.0b013e318283c83a 23343649

[B29] Albiani DA Hodge WG Pan YI Urton TE Clarke WN Tranexamic acid in the treatment of pediatric traumatic hyphema Can J Opthalmol 2008 43 428 431 10.3129/i08-066 18711456

[B30] Kauvar DS Lefering R Wade CE Impact of hemorrhage on trauma outcome: an overview of epidemiology, clinical presentations, and therapeutic considerations J Trauma 2006 60 S3 S11 10.1097/01.ta.0000199961.02677.19 16763478

[B31] Mitra B Cameron PA Mori A Fitzgerald M Acute coagulopathy and early deaths post major trauma Injury 2012 43 22 25 10.1016/j.injury.2010.10.015 21145056

[B32] Talving P Lustenberger T Lam L Inaba K Mohseni S Plurad D Green DJ Demetriades D Coagulopathy after isolated severe traumatic brain injury in children J Trauma 2011 71 1205 1210 10.1097/TA.0b013e31820d151d 21427617

[B33] Peiniger S Nienaber U Lefering R Braun M Wafaisade A Borgman MA Spinella PC Maegele M Trauma Registry of the Deutsche Gesellschaft für Unfallchirurgie Glasgow Coma Scale as a predictor for hemocoagulative disorders after blunt pediatric traumatic brain injury Pediatr Crit Care Med 2012 13 455 460 10.1097/PCC.0b013e31823893c5 22422166

[B34] Whittaker BW Christiaans SC Altice JL Chen MK Bartolucci AA Morgan CJ Kerby JD Pittet JF Early coagulopathy is an independent predictor of mortality in children after severe trauma Shock 2013 39 421 426 10.1097/SHK.0b013e31828e08cb 23591559 PMC3689548

[B35] Holmes JF Goodwin HC Land C Kuppermann N Coagulation testing in pediatric blunt trauma patients Pediatr Emerg Care 2001 17 324 328 10.1097/00006565-200110000-00002 11673707

[B36] Hendrickson JE Shaz BH Pereira G Atkins E Johnson KK Bao G Easley KA Josephson CD Coagulopathy is prevalent and associated with adverse outcomes in transfused pediatric trauma patients J Pediatr 2012 160 204 209 10.1016/j.jpeds.2011.08.019 21925679

[B37] Patregnani JT Borgman MA Maegele M Wade CE Blackbourne LH Spinella PC Coagulopathy and shock on admission is associated with mortality for children with traumatic injuries at combat support hospitals Pediatr Crit Care Med 2012 13 273 277 10.1097/PCC.0b013e31822f1727 21926654

[B38] Vogel AM Radwin ZA Cox CS Jr Cotton BA Admission rapid thrombelastography delivers real-time ‘actionable’ data in pediatric trauma J Ped Surg 2013 48 1371 1376 10.1016/j.jpedsurg.2013.03.036 23845632

[B39] Royal College of Paediatrics and Child Health Evidence statement. Major trauma and the use of tranexamic acid in children November 2012 http://www.rcpch.ac.uk/system/files/protected/page/121112_TXA%20evidence%20statement_final%20v2.pdf

[B40] Harvey V Perrone J Kim P Does the use of tranexamic acid improve trauma mortality? Ann Emerg Med 2013 pii S0196-0644(13)01341-3 24095056 10.1016/j.annemergmed.2013.08.028

